# Characterization of a Unique Nuragic Bronze *Navicella* with a Combination of X-ray Fluorescence Spectrometry and Monte Carlo Simulation

**DOI:** 10.3390/ma16237345

**Published:** 2023-11-25

**Authors:** Marta Porcaro, Sergio Lins, Anna Depalmas, Rosario Maria Anzalone, Roberta Iannaccone, Antonio Brunetti

**Affiliations:** 1 Department of Earth Sciences, Sapienza University of Rome, 00185 Rome, Italy; 2 Department of Basic and Applied Sciences for Engineering, Sapienza University of Rome, 00161 Rome, Italy; sergio.lins@uniroma1.it; 3 DUMAS Department, University of Sassari, 07100 Sassari, Italy; depalmas@uniss.it; 4 Musei Reali di Torino, 10122 Torino, Italy; rosariomaria.anzalone@cultura.gov.it; 5 Chemistry, Physics, Mathematics and Natural Science Department, University of Sassari, 07100 Sassari, Italy; roberta.iannaccone@ispc.cnr.it; 6 Biomedical Sciences Department, University of Sassari, 07100 Sassari, Italy

**Keywords:** Nuragic bronze, cultural heritage, MC-XRF, Raman spectroscopy, bronze corrosion

## Abstract

This paper describes the results obtained from an archaeometric study of a bronze Nuragic small boat model (Sardinia, Italy) dating from the Early Iron Age (presumably 9th–7th centuries BC). The artifact comes from an unknown location in Sardinia and is one of the objects that came to the Museum of Turin in the 19th century. This model is of particular interest as it is a unique Nuragic boat model containing a human figure among its decorations. The artifact is kept in the collection of the Royal Museum of Turin (Italy) and is a typical example of Sardinian manufacture from the Early Iron Age. This study was carried out using a combination of non-invasive techniques with energy dispersive X-ray fluorescence spectrometry (ED-XRF) integrated with Monte Carlo (MC) simulations and Raman spectroscopy, which allowed the characterization of the alloy of the artifact.

## 1. Introduction

Between the 17th and 7th centuries B.C., a period comprehending the Nuragic Age (17th–13th centuries B.C.) and the Final Bronze and Early Iron Ages (12th–7th centuries B.C.), Sardinia was characterized by a significant production of bronze artifacts, with a progressive increase in the number and categories of objects produced, reaching its peak in the Early Iron Age (mid-10th to 7th century BC) [1]. Among the rich Nuragic metallurgy production, small boat models, also known as *navicelle*, hold a prominent place. They are small bronze sculptures mainly characterized by a basin reproducing the hull of a boat and a mast. More than one hundred and fifty of these bronze artifacts are known to date [1].

The *navicelle* likely served as votive lamps inside sanctuaries and shrines where they were placed as votive offerings of prestige and importance. Most of their production took place in the Early Iron Age between the 10th and 7th centuries B.C. However, some chronological aspects are still uncertain due to the scarcity of finds in Sardinia from reliable stratigraphic contexts. Discoveries outside the island range from between the 8th and 7th centuries B.C.

Bronze reproductions of ship models display a high artistic value given the ornamental and descriptive details that enrich the basic model consisting of a vessel hull with an animal protome at the bow.

The *navicelle* were commonly made using the lost wax technique, meaning that the object was molded in a material with high plasticity, which conferred the possibility of rendering finer details. The rudimentary craftsmanship of the Nuragic people—in line with that of most ancient metalworkers—leads us to believe that welding complex parts together to compose the final artifact was a difficult task. It is believed that most of the time parts of the model were cast separately in wax and later assembled. There is evidence that, in some cases, parts were cast separately (hull, protome, and decorations) and later joined by making a new casting chamber [1].

**Figure 1:**
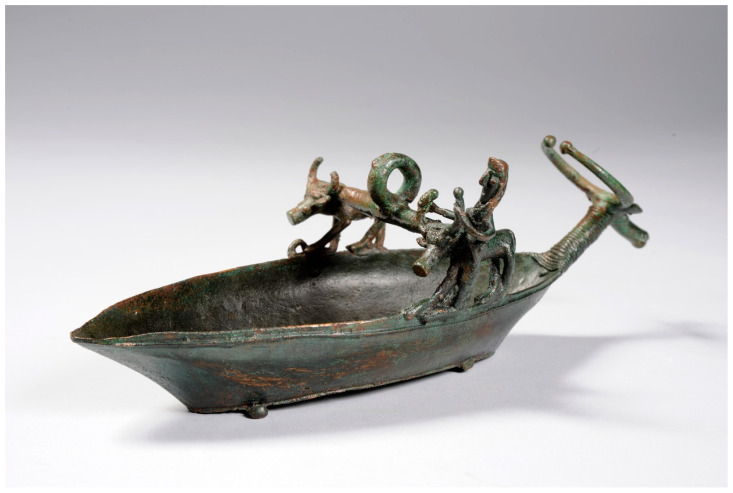
*Navicella* (7990) with plowing scene. “©MiC—Musei Reali, Palazzo Reale”.

The *navicella* (inventory number 7990) (Figure 1) presented in this case study comes from the Royal Collection of the House of Savoy, a collection that comprises numerous artifacts of Sardinian origin now preserved in the Royal Museum of Turin. This object is likely the most representative model from the Sardinian collection with a high level of detail and a fine execution technique including the representation of a plowing scene over the boat’s gunwale. Such qualities emphasize its symbolic and cultic value.

The artifact features a transverse bridge supported by two bovine figures, one on each side of the boat. In addition, we observe one unique case in this category of bronzes (*navicelle*)—the presence of a male figure with bare feet in the act of striding. The figure of the plowman with a dagger on his chest can be traced back to a definite celebratory intent of the young male’s sexual maturity and thus his full-fledged entry into the social group. The sculpture enriches the artifact with meaning and evocative symbolic value, making it a particularly valuable liturgical ornament. 

Besides this archaeological study, it is also important to characterize the sample from the point of view of the composition of the alloy, including the patina (corrosion) layer, if possible, which can give us information about the technology used to create these artifacts and their burial environment [2]. The latter information is often very important since the majority of the Nuragic bronzes have been found outside the original burial site which remains unknown, such as in the case of the boat studied here.

The use of archaeometric techniques to better understand the metallurgical technologies and skills possessed by a group or population has become a staple in the field of cultural heritage diagnostics. 

However, due to the preciousness and sometimes uniqueness of the samples, as well as the increasingly frequent impossibility of taking them out of museums, the characterization of cultural assets suffers from significant limitations. For this reason, the use of portable equipment and non-destructive techniques is effectively mandatory. The principal and most widespread technique is portable X-ray fluorescence (pXRF). X-ray fluorescence is based on the interaction of X-ray radiation, generally emitted by a tube in the case of portable instrumentation, with the object under examination. For the energy range normally available in pXRF, up to 50 keV, the possible interactions are photoelectric, coherent (Rayleigh) scattering, and incoherent (Compton) scattering. The photoelectric interaction can be followed by the emission of a photon, called fluorescence, or an electron (Auger effect). The latter is not relevant here because the electron is rapidly reabsorbed within the sample without producing detectable effects. The emission of fluorescence photons, on the other hand, is the most important effect for sample characterization. In fact, the energy of the emitted photons is specific to a particular chemical element and therefore represents a revealing signal of the presence of that element. Moreover, the number of photons emitted at a specific fluorescence energy is roughly proportional to the concentration of that chemical element.

The other significant effect is Compton scattering. It forms an irregular background under the peaks, encompassing all the energies emitted by the radiogenic tube. While the background is not directly attributable to a specific element, it can be used to estimate the equivalent composition of those chemical elements present in the sample but not producing detectable fluorescence photons under typical experimental conditions used in pXRF, i.e., in air. This is the case for light elements present in the case of metals, in the corrosion patina, and in any protective coatings applied to the artifact to block corrosive processes, such as, for example, Paraloid or similar protectants. In summary, the final result of this interaction is represented by a histogram of the number of photons as a function of their energy, i.e., a set of fluorescence peaks superimposed on a continuous background. From this histogram, a qualitative estimate of the sample’s composition can be obtained by observing the relative amplitudes of the peaks. However, the characterization of a bronze artifact requires an accurate estimate of the alloy’s composition, which can be used both to help determine its origin and the manufacturing techniques [2]. There are various approaches to quantifying an XRF spectrum, ranging from calibration scales obtained from measurements on certified samples under the same measurement conditions [3,4,5,6,7] to general techniques, among which the most well-known is the Fundamental Parameters Method (FPM) [8,9,10,11]. The latter is based on an iterative algorithm using atomic parameters, such as, for example, the probability, given a certain energy of the excitation photon, of producing a certain fluorescence line. These techniques are very fast but do not work very well on complex samples like ancient bronzes, which are generally representable as a structure with irregular multiple layers, where the innermost layer is the actual alloy, covered by one or more layers of corrosion and possibly a protective layer, although algorithms capable of dealing with multilayers have been developed [11,12]. Furthermore, these algorithms require the extraction of the areas occupied by the peaks by removing the background and performing deconvolution operations to separate any overlapping peaks. Background removal can be performed using different techniques, from peak smoothing to its description using polynomial or more complex functions. In all these cases, a perfect description of the background is often not achieved, which, as a consequence, may lead to an overestimation or underestimation of the areas under the peaks, introducing an error in the determination of the concentrations of the chemical elements. The error will be greater the less intense the peak overlying the background. To overcome this inconvenience, a much more precise, albeit more complex and slower technique than the algorithms described above, can be used. This alternative technique integrates XRF measurements with Monte Carlo simulations of the real experiment [13,14,15,16,17,18,19,20,21]. In principle, the Monte Carlo simulation should reproduce a spectrum similar to the measured one. When this condition is met, the model used for the simulated sample is an excellent representation of the real one. All of this can be achieved without any action on the spectra, such as background removal. We wrote “excellent reproduction” and not “perfect reproduction” of the real sample because, for example, the corrosion layer generally presents local irregularities in thickness, which are not visible, while the Monte Carlo model is represented by regular planes. However, as we will see, and as has been demonstrated in other publications, this is not a problem, allowing quantifications comparable to those obtainable with invasive techniques, potentially more precise [22]. For these reasons, this approach was used in this work and will be described in detail in the next section.

The Monte Carlo simulations, particularly the choice of the initial model for the simulated sample, along with visual inspection that, based on the colors of the corrosion, can provide an initial indication of the composition of the patina, have been complemented by Raman spectroscopy. In fact, the use of Raman spectroscopy for the characterization of corrosion products on metal artifacts is well attested [23,24,25,26] with both portable and benchtop instrumentations, although the low crystallinity degree of the corrosion compounds causes a low Raman scattering signal [25].

## 2. Materials and Methods

In this paper, as mentioned in the previous section, we used a combination of ED-XRF and Monte Carlo simulations (MC). Monte Carlo simulations are based on probabilistic algorithms that try to approach the solution to a specific problem that cannot be solved in any analytical way, mostly due to its dimensionality in terms of number of parameters involved. The areas of application are virtually infinite. However, its use may sometimes be limited by the time required to obtain a significative simulation, where “significative” means, in terms of ED-XRF simulations, obtaining a good statistics spectrum (simulated) comparable to the measured (real) one. To explain this point better, let us remember that the statistical error in each energy channel of the spectrum is equal to the square root of the number of counts (photons) in the same channel. A generic Monte Carlo simulation, although specialized for X-ray interactions with matter and despite incorporating some acceleration techniques known as ‘variance reduction’, may require from several hours to several days to simulate a spectrum with good statistics. This is essentially due to the fact that this type of Monte Carlo simulation also considers interactions that are negligible for the type of experiments and energy considered here, such as simulating the fate (transport) of electrons produced by the interactions of photons with the sample. In order to overcome this limitation, some specialized Monte Carlo codes have been developed [19,20,21]. Here we use the XRMC code, which is based on the *Xraylib* database [21]. This Monte Carlo code allows one to obtain a good-quality spectrum in just a few seconds. An in-depth description of its use for Cultural Heritage samples is extensively described elsewhere [15]. Here we have limited ourselves to just summarizing the protocol applied for the quantification of the chemical species present in the sample.

The measurement protocol can be divided into two phases. The first, which can be considered preparatory to the actual simulations, involves characterizing the system, namely the emission from the X-ray tube, the detector response, and the geometry of the system. Characterizing the emission from the X-ray tube is the most complex. It can be performed using equations approximating the tube’s emission, which are used by many quantification algorithms but are rather imprecise for precise elementary quantification. Alternatively, it can be carried out by measuring the tube’s emission. This latter operation can be conducted either by directly measuring the X-ray tube emission or by measuring the radiation from a known sample. Both approaches have their challenges. The first is that emitting radiation from the tube, even at a minimum current (in our case 5 μA), ‘blinds the tube’, resulting in the loss of many counts when the detector is placed at the intended measurement distance. Alternatively, if placed far away, air attenuation causes the low-energy photons contribution to be irreversibly lost, effectively deteriorating the description of the measurement spectrum from about 2 keV and below, resulting in the loss, in our case (X-ray tube with a rhodium anode), of the L fluorescence lines emitted by the anode. The second method is based on Compton scattering, which effectively reproduces a spectrum shifted in a way dependent on the energy of the photons emitted by the tube. This is difficult to correct precisely because of the nonlinear dependence on photon energy. We chose to use the first method, where the obtained spectrum was then used for measurements and simulations on reference samples. The differences between the two spectra, real and simulated, were then used to iteratively correct the estimated spectrum until obtaining a perfect reproduction. The spectrum of the tube thus obtained is what we then used for the simulations. This procedure is very lengthy and is valid only for the energy of the tube used, but, in our opinion, it is the most realistic approach. There is also the possibility of simulating the operation of the X-ray tube using one of the ‘complete’ Monte Carlo methods described above, but this approach depends on the availability of technical data on the tube used, which manufacturers often do not provide.

The response of the detector is relatively simple, being based on always-known data, namely the type and thickness of the detector’s protective window, and the type and thickness of the detector itself. Slightly more complex is characterizing the detector’s response in terms of the ‘deformation’ of the fluorescence peak. In fact, the emitted peak, and thus reproduced by the Monte Carlo simulation, is actually a line at precise energy, but the energy resolution of the detector distorts it, reproducing an almost Gaussian curve. In our case, we adapted the model described by He et al. [27]. There is nothing particular or complicated in the description of the geometry, which is essentially a description of the position of the tube–sample–detector in a three-dimensional space. After these mandatory steps, the following protocol is used:

- A simulation of the real measurement;
- Comparison between the simulated and measured spectra;
- If any differences above a preselected threshold (chi-squared test) are observed, the composition and/or the structure of the simulated sample is updated, and the simulation/comparison is repeated.

This iteration will stop only when the differences are below the threshold established beforehand. It is important to note that the XRF information that can be obtained concerns the first 100 to 300 µm in depth of the sample—at most. It depends on characteristics such as the composition of the object and/or its density. In a highly corroded artifact (elevated corrosion thickness), for example, it is extremely difficult to obtain data regarding the original alloy, as X-rays produced at such a depth and with sufficient energy to reach the detector are statistically insignificant or totally absent. Nevertheless, this has been scarcely observed in the ancient bronzes we have analyzed in the past, and in every case where corrosion layers of a couple hundred microns were present, the Monte Carlo simulations were able to obtain data from the bulk [2].

**Figure 2:**
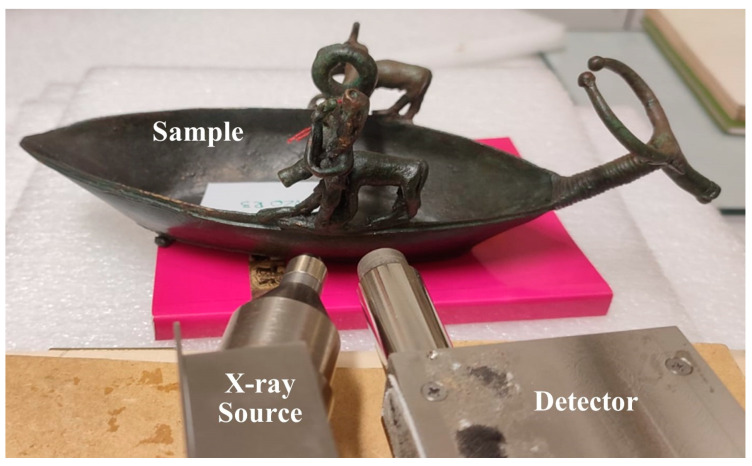
Image of the ED-XRF instrument’s set-up used for the measurement campaign.

The XRF instrument used in this work is a custom portable XRF system equipped with a Mini-X X-ray source with a rhodium anode and one SDD X-123 detector (both manufactured by Amptek Inc. Bedford, MA, USA), observable in Figure 2. The experimental settings were 40 keV and 5–20 µA, with a distance from the sample maintained at about 2 cm. This instrument allows one to change the measurement geometry according to the morphology of the sample, as well as the distance from the sample. This flexibility gives a big advantage with respect to commercial ED-XRF apparatus—handheld systems, for example—which have a fixed geometry.

With the perspective of a non-invasive campaign, a portable Raman instrumentation was used to investigate the presence and the nature of corrosion compounds on the surface. Raman spectra of the surface were collected by a Bravo Raman portable spectrometer (Bruker Optics, Ettlingen, Germany) equipped with two laser excitations to reduce fluorescence, a CCD detector, and a laser power range < 100 mW. The measuring spot is 10–15 μm and the head with a soft support provides a correct focal distance, with the laser ideally focused when the instruments are working on contact [28,29].

## 3. Results and Discussion

The surface of the artifact studied in this work can be considered a multi-layered structure. The simulation performed showed that the object is formed by the deepest layer (bulk or alloy), onto which one or two corrosion layers are superimposed, on top of which, finally, a protective layer—probably applied during a past restoration campaign—is present. It is worth noting that the XRF technique is a surface-type technique. Therefore, the presence—and especially the thickness—of the corrosion layers is valuable data to help distinguish them from the bulk. Several measurements have been acquired in order to obtain a meaningful characterization of the alloy and the corrosion layer on the entire object.

In this case, it was possible to discriminate the alloy layer in every point analyzed because the corrosion was not too thick in any spot sampled, probably due to the actions taken during the restoration campaign which removed the ‘coarse’ patina. The bulk material is made of a typical ternary alloy, composed of copper, tin, lead, and a small amount of silver (0.05% to 0.17%). Silver is not unusual in Cu alloy artifacts; its presence in such low percentages indicates that it comes from the ores used for smelting [30,31,32]. All the measured points are reported in Table 1.

Although this bronze is made of a ternary alloy, with an average lead content of 2.6%, the element is not miscible in the Cu/Sn mixture and cannot modify the bronze phases [33]; thus, it is possible to speculate simply on a Cu/Sn ratio with good confidence.

**Figure 3:**
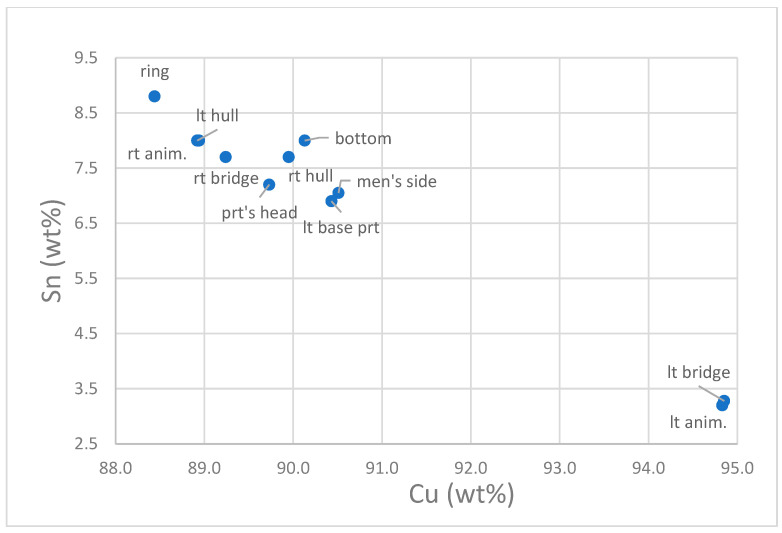
Graphic of the ratio between copper (Cu) and tin (Sn) of measured points.

The percentages of copper vs. tin of each measured point reported in Figure 3 allow us to better figure out the distribution of the ratio between the two main elements.

Observing Figure 3, almost all the points are placed in the upper left side of the graphic, while just two measures appear totally different.

Both the two points were acquired on the left side of the boat, corresponding to the animal and the transverse bridge.

Both areas observable to the naked eye show a different surface color, reflecting the different Cu/Sn ratio. The alloy used for the bovine on the port side and the left portion of the bridge appears to be the same, with a concentration of tin lower than that of the rest of the artifact (around 3.0%) (see Table 1).

The difference ratio between copper and tin for these two measures, placed both on the port side, and the observation of the connection between the hind legs of the animal and the hull suggests that this is a repair or a reworking of the artifact.

The *navicella* is covered with a layer of protective coating with an average thickness of 40 µm. The patina layer appears across the entire surface and suggests a comprehensive intervention to protect the metal as reported in the literature [33,34]. 

Usually, the thickness and composition of the oxide layer can provide information on the type of corrosion that the artifact has undergone over time [35]; despite this, the presence of a past restoration allows us to only speculate on its burial environment and history.

The elements that constitute the corrosion layer derive from both the composition of the alloy and the interaction of the artifact with the environment which it has been in contact with. In fact, exogenous elements such as calcium, sulfur, arsenic, iron, iodine, and potassium, which have been identified on the surface of the artifact, are common to many archaeological finds and can be related to the soil composition of the burial site. 

A superficial enrichment of tin is noticed only in the measurements made on the elements on the starboard side: animal, bridge, human figure, and the hull itself. This type of process called decuprification [33,36,37] is related to the selective dissolution of copper, depending on the corrosivity of the context, and it is the main phenomenon that occurs in type I patina, also called *even surface*. Changes in corrosive conditions can vary the tin content on the surface [38]. In contrast, on the port side, we found a complete destannification of the surface, a loss of tin in the outermost area of the artifact. This process is due to selective corrosion and the subsequent deposition of copper-based products on the surface. Since tin oxide is insoluble, this usually occurs in reducing conditions [39].

**Figure 4:**
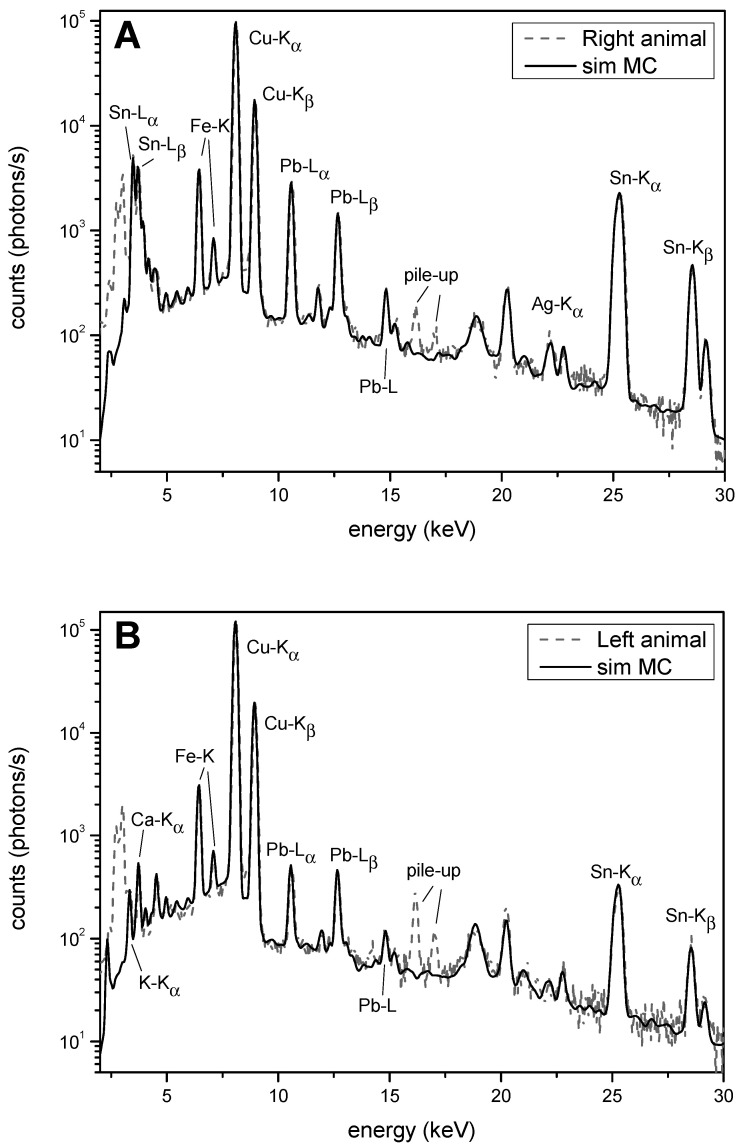
Comparison of the measured spectra (dashed line) and the simulated ones (solid line) of the right (**A**) and left (**B**) animals.

Observing the spectra obtained from the simulation and XRF measurements (Figure 4), the presence of the L lines of tin appears evident only in the spectra obtained from the right-side animal, which indicates, as mentioned above, a greater presence of tin on the surface.

**Figure 5:**
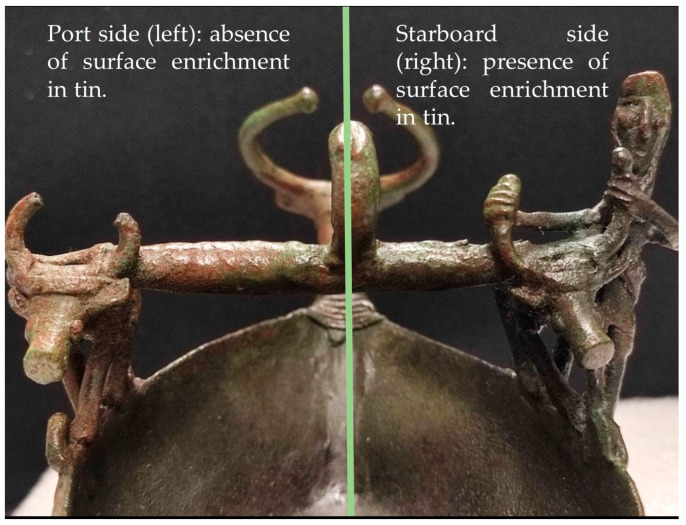
*Navicella* details. Longitudinal division of the artifact based on the color of the surface patina. (**Left side**): reddish patina—area without surface tin enrichment. (**Right side**): blackish-green patina, area with superficial tin enrichment.

It is interesting to note how, even with the naked eye, the *navicella* can be easily divided longitudinally into two different sections based on the color of the surface patina (Figure 5).

**Figure 6:**
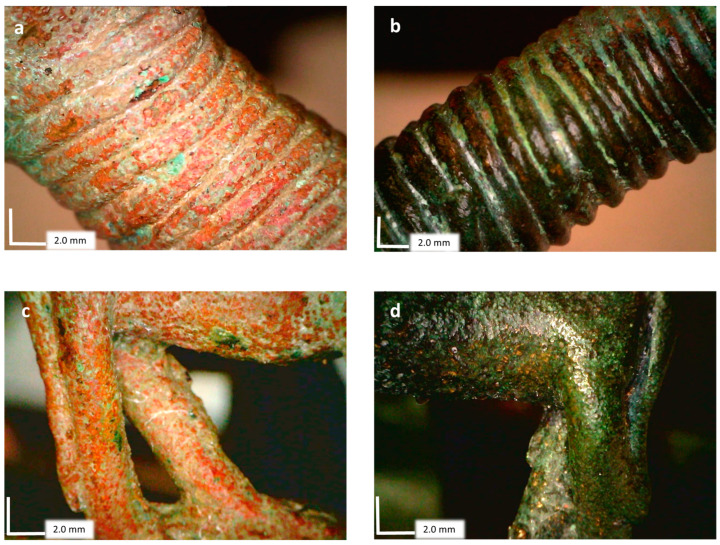
Patina details were observed on Dino-Lite’s portable USB microscope (20×). (**a**) Protome’s neck left side; (**b**) protome’s neck right side; (**c**) left animal hind limbs; (**d**) right animal hind limbs.

The clear distinction between the right and left sides of the artifact (Figure 6) can be associated with a different burial environment which produced different conditions and reactions.

**Figure 7:**
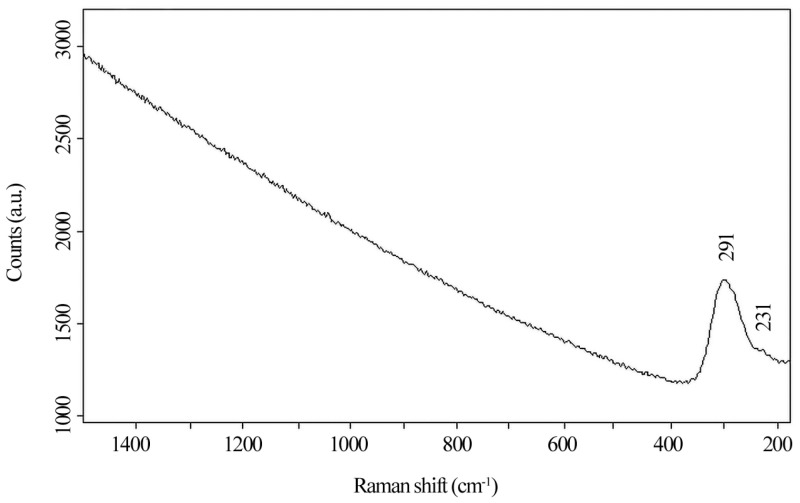
Raman spectrum of a point where tenorite and cuprite peaks are both visible. The peak for cuprite appears as a small shoulder at 231 cm^−1^.

Raman spectroscopy was used as a support to the measures already obtained with ED-XRF, challenging the recognition of the patina’s compounds on *navicella*. Despite the Raman characteristic to also recognize poorly crystalline structures or amorphous compounds [25], all the spectra acquired show only the presence of a peak at 291 cm^−1^ corresponding to poor crystallinity cupric oxide (tenorite). The peak in each spectrum varies in sharpness and intensity and it can be used as an indicator of crystallinity degree together with the red shift of the peak’s center. An attempt to recognize the presence and the type of tin oxides in the starboard side was made, but no evidence of Sn compounds was found. However, this does not mean that they are missing but that, for several reasons, they cannot be recognized with the instrument used [25]. The presence of tenorite can be ascribed to prolonged contact with an alkaline environment or heating process [39,40,41,42]; nevertheless, both oxides (cuprite and tenorite) were probably formed on the surface, but only tenorite was clearly detected except for two analyzed points where a weak shoulder at 231 cm^−1^ referring to cuprite can be seen (Figure 7).

## 4. Conclusions

The ED-XRF technique combined with MC simulations has demonstrated its effectiveness in characterizing artifacts without the necessity of sampling or damaging the object.

The information obtained on the elemental composition of the *navicella* showed that the artifact was made of bronze (a ternary alloy of Cu, Sn, and Pb), with percentages of the main elements consistent with other *navicella* samples already studied in the past. The decorative elements on the port side are made of alloys different to that of the rest of the hull, suggesting a later addition of such elements or a reworking of the object.

In addition, the results obtained on the corrosive layers of the artifact reveal a clear division between the port and starboard sides of the boat. All measurements taken on the right side of the *navicella* indicate a superficial enrichment of tin, while the measurements taken on the left side reveal a complete surface destannification. This heterogeneity was likely caused by different corrosion processes and thus variations in the conservation environment.

Raman spectra supporting the ED-XRF analysis show the presence of tenorite on both sides of the *navicella* and a small amount of cuprite, suggesting prolonged contact with an alkaline environment or heating process in the past.

## Figures and Tables

**Table 1:** Bulk quantitative data of the different regions of interest of *navicella* #7990, expressed in weight percentages (wt%). Only elements with concentrations greater than 0.5 wt% are shown in the table. The error is about 5% of the measured value for the chemical elements with concentrations higher than 1% and 10% for the others.

Area	Copper (Cu wt%)	Tin (Sn wt%)	Lead (Pb wt%)
Right side (hull)	89.9	7.7	2.2
Left side (hull)	88.6	8.0	3.2
Hull bottom	90.1	8.0	1.7
Left animal	94.8	3.2	1.9
Right animal	88.9	8.0	2.9
Left bridge	94.8	3.3	1.8
Right bridge	89.2	7.7	2.9
Side of man	90.5	7.0	2.3
Ring	88.4	8.8	2.6
Left base protome	90.4	6.9	2.5
Head protome	89.7	7.2	2.9

## Data Availability

The data generated are available upon request to the authors.
